# Aging-Related Sarcopenia: Metabolic Characteristics and Therapeutic Strategies

**DOI:** 10.14336/AD.2024.0407

**Published:** 2024-04-07

**Authors:** Yonglian Chen, Jinhui Wu

**Affiliations:** Center of Gerontology and Geriatrics, National Clinical Research Center for Geriatrics, West China Hospital, Sichuan University, Chengdu, 610041, China.

**Keywords:** aging, sarcopenia, skeletal muscle, metabolomics, therapeutics

## Abstract

The proportion of the elderly population is gradually increasing as a result of medical care advances, leading to a subsequent surge in geriatric diseases that significantly impact quality of life and pose a substantial healthcare burden. Sarcopenia, characterized by age-related decline in skeletal muscle mass and quality, affects a considerable portion of older adults, particularly the elderly, and can result in adverse outcomes such as frailty, fractures, bedridden, hospitalization, and even mortality. Skeletal muscle aging is accompanied by underlying metabolic changes. Therefore, elucidating these metabolic profiles and specific mechanisms holds promise for informing prevention and treatment strategies for sarcopenia. This review provides a comprehensive overview of the key metabolites identified in current clinical studies on sarcopenia and their potential pathophysiological alterations in metabolic activity. Besides, we examine potential therapeutic strategies for sarcopenia from a perspective focused on metabolic regulation.

## Introduction

The average life expectancy is progressively rising across the majority of regions. It is projected that by 2050, approximately one-fifth of the global population will consist of individuals aged 60 or above. Aging-related degenerative changes have a profound impact on various organs and systems within the human body, encompassing the nervous system, circulatory system, endocrine system, digestive system, locomotor system, and respiratory system [1-6]. Sarcopenia, a prevalent age-related condition among the elderly [7, 8], is frequently disregarded. Muscular strength and mass peak in the 20s and 30s, subsequently experiencing a gradual decline after the age of 50 [9].

Sarcopenia increases the risk of adverse outcomes such as falls, fractures, bedridden, hospitalization, and even mortality [10]. The estimated cost of comorbid sarcopenia hospitalizations in the US for those over age 40 is approximately $4.04 billion U.S. dollar - a significant increase compared to non-sarcopenic patients [11]. Therefore, early diagnosis and effective interventions for sarcopenia are crucial to alleviate healthcare costs and improve quality of life.

It has been proposed that the primary etiology of sarcopenia is "aging", and thus sarcopenia has been defined as the age-related decline in muscle mass [12]. Various components of skeletal muscle at both macroscopic and microscopic levels undergo alterations with advancing age. It is evident that limb circumference tends to decrease in aging individuals [13], exhibiting a strong association with muscle mass and overall physical performance [13-15]. Histological examinations of the skeletal muscle of the human body have further elucidated aging-related structural changes. For instance, Lexell and colleagues observed a significant reduction in muscle size among older individuals compared to younger ones, accompanied by a decline of ~25% in the number of muscle fibers [16]. A statistical analysis conducted on fiber types revealed a significant reduction in the proportion of type II fast muscle fibers among aging individuals, primarily observed in the peripheral region of the muscle fascicle [17]. Additionally, there was a notable reduction in contractile function specifically within type II muscle fibers, indicating a consistent trend with age-related decreases in mobility and flexibility [18, 19]. Aging skeletal muscles are also characterized by the infiltration of intermuscular adipose tissue, irrespective of changes in body weight [20, 21], accompanied by matrix deposition [22, 23]. This leads to an increase in muscle hardness and disruption of the injury response mechanism. Swelling, increased fusion, and irregular morphology of mitochondria in the extensor digitorum longus muscle have been observed in rodents, where there is widening of the interstitial space between myogenic fibers and increased deposition of matrix [24]. The motor unit, which serves as the fundamental functional unit of skeletal muscle, undergoes dynamic reorganization characterized by reduced neuronal innervation, incomplete regeneration of nerves, and structural alterations at the neuromuscular junctions [7]. However, the metabolic changes associated with these structural and functional modifications remain uncertain.

**Figure 1.**
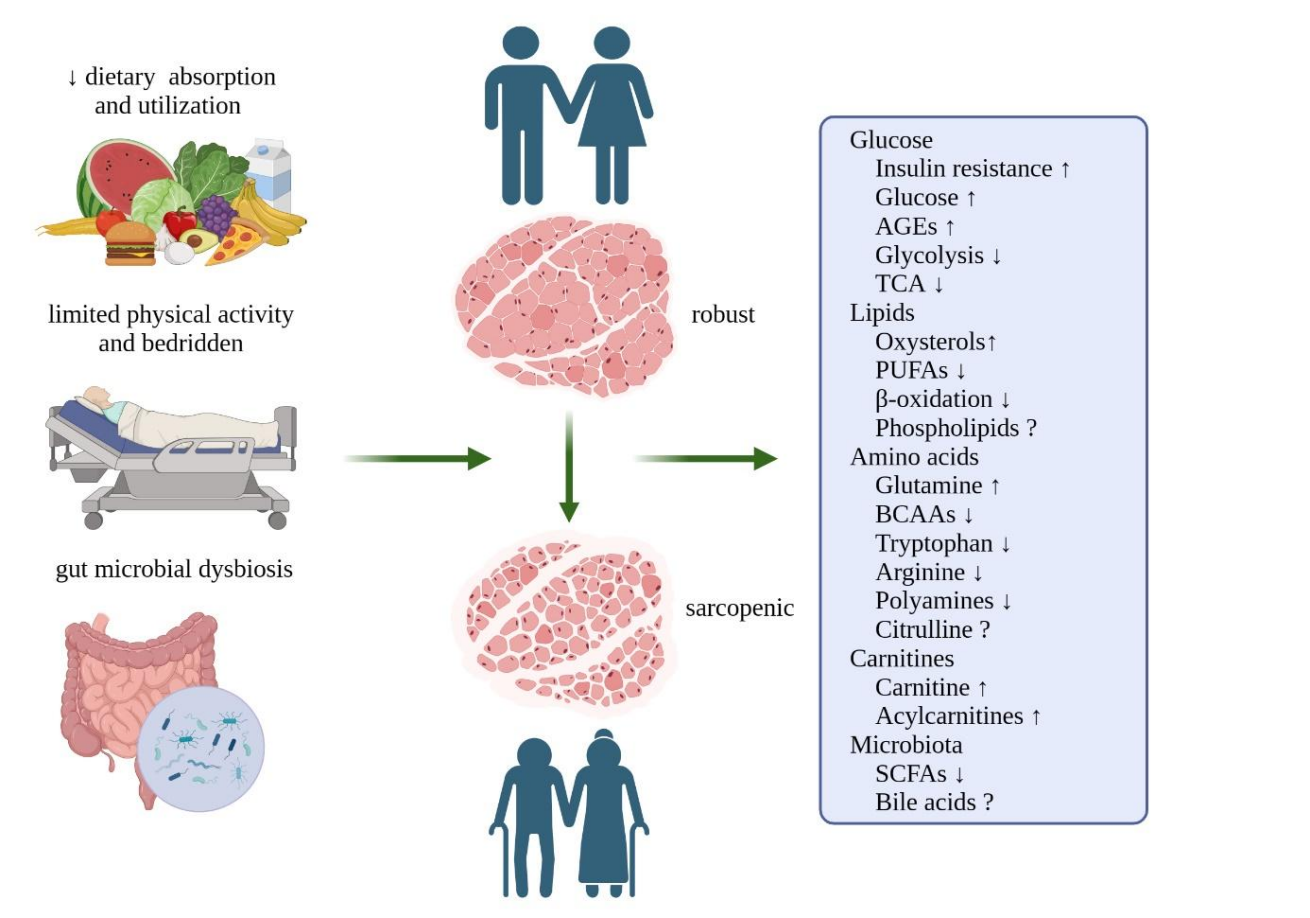
**Key metabolic changes associated with sarcopenia (created with BioRender.com)**. With the progression of aging, there is a gradual decline in muscle quality and subsequent compromised mobility attributed to diminished gastrointestinal tract digestion and absorption, reduced physical activity or bedridden, as well as gut microbial dysbiosis. This intricate process is accompanied by numerous metabolic alterations that can serve as potential indicators for early sarcopenia diagnosis while also offering insights into disease pathogenesis and facilitating the development of therapeutic strategies. Abbreviations: AGE: advanced glycosylation end product; TCA: tricarboxylic acid cycle; PUFA: polyunsaturated fatty acid; BCAA: branched chain amino acid; SCFA: short-chain fatty acid. ↓: down-regulated; ↑: up-regulated; ?: inconsistent findings.

Skeletal muscle contractile activity necessitates substantial amounts of energetic substrates. In the elderly population, a common observation is the presence of decreased or restricted physical activity, which subsequently triggers remodeling and adaptation of metabolic activity in skeletal muscles [25, 26]. Furthermore, the process of aging leads to a decline in the absorption and utilization of essential nutrients such as carbohydrates, fats, and proteins among older individuals (Fig. 1). This may further disrupt skeletal muscle metabolic homeostasis due to inadequate metabolism or decreased clearance of detrimental byproducts [27]. For instance, an inflammatory milieu diminishes glucose uptake by skeletal muscle, exacerbating insulin resistance and potentially leading to the development of diabetes. Moreover, impaired insulin signaling may contribute to proteolysis and anabolic resistance. Furthermore, hyperglycemia can exacerbate the impairment of skeletal muscle integrity by inducing microvascular and neural damage through advanced glycosylation end products (AGEs) [28, 29].

Metabolomics has emerged as a powerful tool for comprehensively exploring the intricate metabolic landscape within an organism, offering novel insights into physiological phenotypes and pathophysiological mechanisms of diseases through the lens of metabolites [30]. Given its significant contribution to approximately 40% of total body mass, skeletal muscle exhibits dynamic and complex metabolic activities [31], with metabolites serving as valuable indicators reflecting fluctuations in metabolism. In recent years, there has been a growing emphasis on conducting metabolomics studies using biological tissues or bodily fluids to identify more closely associated biomarkers for sarcopenia. These biomarkers provide crucial insights for further elucidating the underlying pathogenesis of this condition. Therefore, this review aims to explore the characteristics and potential intervention strategies associated with sarcopenia from a perspective encompassing aging and metabolic factors.

## Amino acids

### Branched chain amino acids

Amino acids, the fundamental constituents of proteins, play a crucial role in maintaining protein homeostasis within skeletal muscle [32]. It has long been recognized that specific types of amino acids in the bloodstream of patients with malignant tumors undergo significant alterations to support the rapid proliferation of tumor cells [33-35]. Among the metabolic changes associated with sarcopenia, particular emphasis has been placed on modifications in amino acid metabolism. For instance, previous studies have indicated that sarcopenia patients exhibit lower concentrations of branched-chain amino acids (leucine, isoleucine, and valine) in their circulation compared to non-sarcopenia controls [36-42] (Table 1). Additionally, these studies have shown a positive correlation between the levels of branched-chain amino acids and skeletal muscle performance/muscle volume. These results suggest that alterations in branched-chain amino acid metabolism may play a significant role in the development of sarcopenia. However, a separate subset of studies has produced contrasting findings. In elderly individuals with chronic activity restrictions, there is an inverse association between branched-chain amino acids and muscle mass [43]. Furthermore, a case-control study revealed that participants with low muscle quality exhibited elevated levels of leucine and isoleucine [44]. It is noteworthy to mention that circulating branched-chain amino acids also exhibit a significant correlation with insulin sensitivity [45]. Actually, branched-chain amino acids account for approximately 35% of the total content of muscle protein and serve as important complements to the energy source of skeletal muscle [46]. The catabolism of these amino acids involves two significant enzymes, namely branched-chain aminotransferase and branched-chain α-ketoacid dehydrogenase. These enzymes are responsible for transamination and oxidative decarboxylation reactions, respectively, which provide ketone bodies and gluconeogenic substrates to skeletal muscles [46]. The resulting products enter the tricarboxylic acid cycle for energy production, subsequently leading to the synthesis of glutamine and alanine [47]. Unfortunately, there is a scarcity of studies that have investigated alterations in the activity or quantity of branched-chain amino acid hydrolase in the skeletal muscle of individuals with sarcopenia. Conversely, a series of studies have observed enhancements in muscle quality following supplementation with branched-chain amino acids or isolated leucine [48]. Correspondingly, the expression of mTORC1 and its downstream effectors, such as eukaryotic initiation factor 4E-binding protein 1 (4E-BP1) and p70 S6 kinase 1 (p70S6K1), exhibited upregulation [49], indicating enhanced protein synthesis. In patients with sarcopenia, disturbances in circulating amino acids may not solely arise from altered skeletal muscle metabolism since multiple disorders commonly coexist in the elderly population. Nevertheless, the findings from this interventional study underscore the significance of these essential amino acids and advocate for further mechanistic investigations to unravel the pathogenesis of sarcopenia.

### Tryptophan

Another important amino acid, tryptophan, appears to have a distinct role in sarcopenia and is currently being extensively investigated. For instance, multiple studies have consistently demonstrated that patients with sarcopenia exhibit significantly reduced levels of tryptophan in their circulation compared to non-sarcopenic individuals [38, 39, 42, 47, 50-53] (Table 1). Additionally, there is a positive correlation between tryptophan levels and muscle mass [42]. In contrast, elevated levels of tryptophan and kynurenine have been observed in skeletal muscle specimens from healthy older individuals compared to younger individuals [54]. This suggests that age and disease can alter the metabolism of tryptophan, potentially offering beneficial effects on skeletal muscle. Following the absorption of exogenous tryptophan from the intestine, it undergoes primary metabolization through three major pathways: the Kyn pathway, 5-HT pathway, and indole pathway [55]. These pathways play distinct roles in metabolism, the nervous system, and inflammatory processes [56], with the hepatic Kyn pathway serving as the predominant metabolic pathway. In reality, the majority of digested and absorbed tryptophan undergoes catabolic metabolism, leading to the production of various biologically active molecules such as serotonin, kynurenines, NAD+, and indoles [56]. Serum tryptophan levels were significantly lower in lymphoma sarcopenic patients compared to the non-sarcopenic group [52]. The proliferation and differentiation of cultured C2C12 cells are significantly hindered in the absence of tryptophan [52]. A diet deficient in tryptophan profoundly alters the amino acid metabolic profile in mouse skeletal muscle, potentially as a compensatory response to tryptophan deficiency, highlighting the essentiality of tryptophan for normal muscular cell activities [52]. After a 12-week exercise regimen, Allison and colleagues observed a significant upregulation in the gene expression of PGC-1α, PPAR-α, PPAR-δ, and kynurenine aminotransferase (KAT) within the skeletal muscles of elderly individuals [57]. All of these findings suggest that the augmented activity in tryptophan metabolism may play a role in the exercise-induced enhancement of skeletal muscle performance. However, further investigation is still required to elucidate the specific mechanisms implicated. Simultaneously, the kynurenine levels in skeletal muscle of elderly individuals increase, while in sarcopenia patients' serum, the kynurenine/tryptophan ratio exhibits a significant negative correlation with muscle mass [42, 54]. This phenomenon can possibly be attributed to the enhanced activity of indoleamine 2,3-dioxygenase, an enzyme involved in tryptophan catabolism [58], due to inflammatory aging.

**Table 1:** Summary of characteristic metabolites in clinical studies of sarcopenia.

PMID	Year	Methods	Specimen	Sample size^&^	Key findings	Specific comorbidity	Diagnostic criteria
**37767786**	2023	NMR	Serum	78/116	Deoxycholic acid, lithocholic acid, ratio of deoxycholic acid to cholic acid, ratio of lithocholic acid to chenodeoxycholic acid, ratio of 12 alpha-hydroxylated to non-12 alpha-hydroxylated BAs↑^$^; Val, acetate↓	Cirrhosis	EWGSOP1
**29464940**	2018	GC-MS /CE-MS /LC-MS	Plasma	8/15	Trp, Thr, indolelactic acid, glycerophospholipids, lysophosphatidylcholines (O-16:0), lysophosphatidylcholines (20:3)sn-1, sphingolipids ↓; cortisol ↑	Cancer	Involuntary weight loss of 5% or more within the previous 6 months
**30404172**	2018	LC-MS	Serum	38/68	Asn, Asp, citrulline, ethanolamine, Glu, sarcosine, taurine ↑; α-aminobutyric acid, Met ↓	Frailty	FNIH
**34636159**	2021	LC-MS	Serum	18/54	Arg, citrulline, His ↓; ornithine, carnitine↑	Cancer	Cachectic cancer patients
**30137216**	2019	LC-MS	Plasma	12/28	Oxylipins derived from EPA and DHA↑	ROOF cohort	Decreased ALM (<-1 %/yr), stable ALM (>-1 and < 0%/yr)
**37438737**	2023	LC-MS	Plasma	30/76	Citrulline, Pro, Ser, Glu↑; Arg, Asn, Phe, Ser, Lys, Gln, Thr ↓	-	AWGS 2019
**29788121**	2019	LC-MS	Plasma	504	LPC 17:0, LPC 18:1, LPC 18:2 (+) with gait speed^*^; SM 16:1, SM 18:0, SM 18:1, PC diacyl (aa) 32:3 (-) with gait speed	-	Gait speed
**32468656**	2020	^31^P-MRS /GC-MS /LC-MS	Skeletal muscle	7/24	Phosphodiester, phosphatidylcholine, phosphatidylethanolamine, phosphatidylglycerol↑; phosphocreatine ↓	-	EWGSOP1
**34492634**	2021	LC-MS	Whole blood	6/19	Aspartate↑; acetyl-carnitine, dimethyl-proline, Phe, dimethyl-arginine, N1-methyl-histidine, isovaleryl-carnitine, myo-inositol, creatinine, pantothenate, hypoxanthine, dimethyl-guanosine, N1-methyl-adenosine, 2-oxoglutarate, pentose-phosphate, succinate, N-acetyl-glutamate, quinolinic acid,4-guanidinobutanoate, N1-methyl-guanosine, trimethyl-tyrosine, cis-aconitate ↓	-	AWGS 2014
**32677363**	2020	-	Plasma	20	Methylmalonate,3-hydroxybutyrylcarnitine,1-stearoyl-2-linoleoyl-GPI (18:0/18:2),1-stearoyl-2-linoleoyl-GPC(18:0/18:2), 1,2-dilinoleoyl-GPC(18:2/18:2), N-acetyl-1-methylhistidine, N-acetylcitrulline, N-acetylglutamine, N-acetylphenylalanine, 3-methylglutarylcarnitine, N-acetylarginine,glutarylcarnitine(C5-DC),N-acetylleucine,hydroxyasparagine,N-acetylserine,5,6-dihydrouridine, orotidine, dihydroorotate↑; 1-(1-enyl-palmitoyl)-2-linoleoyl-GPE(P-16:0/18:2), gamma-glutamylmethionine, cytidine↓	Patients underwent aortic surgery	Loss of >10% of the cross-sectional area of the rectus femoris, (RF_CSA_) + grip strength, quadriceps strength
**37127296**	2023	LC-MS	Plasma /muscle	71/142	Oleoyl ethanolamide, decanoylcarnitine,1-stearoylglycerol, stearic acid (+) with ASM; stearamide, oleamide, palmitamide, docosanamide, stearoyl ethanolamide (-) with ASM	-	AWGS 2019
**25898920**	2016	LC-MS	Plasma	3953	Urate, glutamate, mannose, gamma-glutamylleucine, gamma-glutamylvaline, Val, Ile, Leu, kynurenine (+) with ALM	-	-
**35352111**	2022	LC-MS	Serum	430	1-aminocyclopropane-1-carboxylate, arecoline, creatinine, dihydrothymine, galactonic acid, glycyl-Leu, imidazole, L-carnitine, L-leucyl-L-proline, L-threonine, ornithine(+) with ALM, ALM/BMI; betaine, glycolithocholic acid, PC (16:0/16:0), Phe-Pro, stearic acid (-) with ALM, ALM/BMI	Early postmenopausal women	-
**33410783**	2020	NMR /LC-MS	Plasma	153	Glutamate, alpha-aminoadipate (+) with SMI; citrulline/ornithine (-) with SMI	-	-
**29030163**	2017	LC-MS	Serum	73	Gln, Ser, glycoursodeoxycholate, Ala, biliverdin, Gly, xanthine, deoxycarnitine, ergothioneine, cortisone, Asn, 7-methylguanine, trans-urocanate, 7-α-hydroxy-3-oxo-4-cholestenoate (+) with muscle composition; mannitol, 2-hydroxyisobutyrate, urea, butyrylcarnitine, pseudouridine, N-acetylthreonine, tiglyl carnitine, erythronate, 4-acetamidobutanoate, C-glycosyltryptophan, erythritol, glutaroyl carnitine, phenylacetylglutamine, dimethylglycine, 5α-androstan-3β, 17beta-diol disulfate, 2-methylbutyroylcarnitine, indolelactate, N-acetylalanine, methylglutarylcarnitine, octanoylcarnitine, myo-inositol, arabitol, arabonate, isobutrylcarnitine, decanoylcarnitine, N-formylmethionine, acetylcarnitine, phenol sulfate, N1-methyl-2-pyridone-5-carboxamide, glucuronate, *Trans*-4-hydroxyproline, laurylcarnitine, N-acetylmethionine, cis-4-decenoyl carnitine, hexanoylcarnitine, symmetric dimethylarginine, N6-acetyllysine, tetradecanedioate, 1,5-anhydroglucitol, epiandrosterone sulfate, threitol, 1-methylurate, androsterone sulfate, N-acetylserine, N1-methyladenosine(-) with muscle composition	Absence of structured exercise during the previous 6 months	Muscle composition indicated by the ratio between normal density with low density thigh muscle cross sectional area
**36658632**	2023	LC-MS	Plasma	8/22	Citrulline, NEFA 26:2, succinic acid, NEFA 24:2, cholic acid, fumaric acid, NEFA 16:3, NEFA 26:1, dicarboxylic acylcarnitines: C8/C6/C10/C7-DC, acylcarnitines: C6-OH/C10:1 (+) with sarcopenia; tauro- cholic acid 3-sulfate (-) with sarcopenia	Hip fracture patients project to surgery	EWGSOP2
**35155500**	2022	LC-MS	Serum	52/246	LPC16:0, LPC18:2, Trp, valeryl-L-carnitine ↓	Frailty	AWGS 2019
**35337292**	2022	LC-MS	Serum	65/246	Ile, Leu, BCAA, Glu, Trp, LPC18:2 (+) with sarcopenia composition; Gly, acylcarnitines: C5/C6/C8/C10/C12/C14 (-) with sarcopenia composition	-	AWGS 2019
**33924750**	2021	LC-MS	Serum	237/729	2-hydroxybutyrate, 4-methyl-2-oxopentanoate, Val, Ile, Leu, Trp, taurine, cystine↓	-	AWGS 2014
**27029859**	2016	LC-MS	Plasma	79/158	Leu, Ile, Trp, serotonin, methionine↑; putrescine, PC diacyl (aa)C32:2, C34:3, C34:4, C42:4, PC acyl-alkyl (ae) C34:1, C38:1, C38:2 C38:3, C40:2, C40:3, C40:4, C40:5, C42:1, C42:3, C42:4, C42:5, C44:3, C44:4, lysoPC acyl (a) C16:1, C18:1, C18:2↓	-	Ratio of the maximum quadriceps strength to thigh CT-scan cross-sectional muscle area
**37242283**	2023	GC-MS	Plasma	27/99	Leu, Ser, Asp, Glu↓	Diabetes	AWGS 2019
**33217429**	2021	LC-MS	Plasma	121	Short-chain dicarboxylic, hydroxylated acylcarnitines (-) with 18-month decline in HGS	-	Grip strength
**32244785**	2020	LC-MS	Serum	54	Trp ↓	Diffuse large B-cell lymphoma	L3 skeletal muscle index
**37243418**	2023	NMR	Urine	90	Dimethylglycine (+) with ALMI; oxoisovalerate, isobutyric acid (-) with ALMI	Rheumatoid arthritis	-
**34498155**	2021	LC-MS	Plasma	20/41	Hexyl furoate, retinol acetate, 4-hydroxy-16,18-tritriacontanedione, arachidonic acid, dihomo-alpha-linolenic acid, tyramine-O-sulfate, 3-(tert-butyl)-N-(2,3-dihydro-1-benzofuran5-ylmethyl)-1-methyl-1H-pyrazole-5-carboxamide, 12-hydroxystearic acid, 1-meadoyl-glycero-3-phosphate, 2,3-dinor-6-oxoprostaglandin F1 alpha, 4-undecylbenzenesulfonic acid↑; theophylline, 15-hydroxypentadecanoic acid, docosatetraenoic acid, alpha-pyrrolidinopropiophenone, Boc-D-asparagine, ethyl eicosapentaenoic acid, glycidyl stearate, (11Z,14Z)-eicosadienoic acid, linoleamide, mebutamate, ribothymidine, 2,3-dihydroxypropyl stearate, spiroxamine, methyl stearate, keratan sulfate I, 16(R)-HETE, 1-O-octadecylglycerol-3-phosphatidylcholine↓	-	EWGSOP1
**35963453**	2022	CE-MS	Plasma	10/20	Ala, homocitrulline, N-acetylserine, gluconic acid, N-acetylalanine, Pro, sulfotyrosine ↑; 4-methyl-2-oxovaleric acid, 3-methyl-2-oxovaleric acid, Trp ↓	-	AWGS 2019
**37033246**	2023	LC-MS	Serum	10/20	Pentadecanoic acid, 5'-methylthioadenosine, N,N-dimethylarginine, glutamine ↑; isoxanthohumol↓	Diabetes	AWGS 2014
**28934309**	2017	LC-MS	Plasma	28/160	Pro, Gln↑; His, Trp↓	-	AWGS 2014
**34939349**	2022	LC-MS	Plasma	24/48	Traumatic acid↑	-	6.76kg/m2 for men and 5.28 kg/m2 for women
**34515116**	2022	LC-MS	Serum	2610	Va, Phe, Glu, Tyr (+) with sarcopenia; Met, Gln (-) with sarcopenia	-	AWGS 2019
**30318485**	2018	LC-MS	Serum	136	Methyl β-D-galactoside, pipecolic acid (+) with muscle strength and mass; aspartate, Glu, 12(S)-HETRE, 12(S)-HETE, arachidonic acid, glycerophosphocholine, phenylalanyl-threonine (-) with muscle strength and mass	-	-
**35235538**	2022	LC-MS	Serum	174	N-acetyl-L-aspartic acid (+) with muscle strength; Glu, carnosine (-) with muscle strength	-	-
**37694554**	2023	LC-MS	Serum	92/279	Leu, Lys, Met, Phe, Thr, Trp, Val, AABA, β-alanine, nicotinamide-to-quinolinic acid ratio (+) with muscle mass; 3-hydroxykynurenine, 3-hydroxykynurenine ratio, Hydroxykynurenine-to-xanthurenic acid ratio, kynurenine-to-tryptophan ratio, quinolinic acid, SDMA, 4-alpha-hydroxy-5-methyl-tetrahydrofolate (-) with muscle mass;	-	D3 Cr dilution skeletal muscle mass
**37993772**	2023	LC-MS	Plasma	63/120	Acylcarnitines: C0/C4/C6/C4:1-DC/C8/C10/C10:1/C12/C14/C14:1/C14:1-OH/C14:2/C16/ C16:1/C16:1-OH/C16:2/C16:2-OH/C18:1/C18:1-OH/C18:2, Arg, citrulline, kynurenine, putrescine, SDMA, PC acyl-alkyl (ae) C44:5, PC acyl-alkyl (ae) C44:6↑; C4/Cr, kynurenine (+) with sarcopenia	-	AWGS 2019
**36875836**	2023	GC-MS	Plasma	20/40	L-kynurenine, inosine-5'-monophosphate, phosphoglycolic acid, D-fructose-6-phosphate, O-phosphoethanolamine, pyrophosphate, N-acetylornithine, trehalose-6-phosphate, uridine 5'-monophosphate, D-ribulose 5-phosphate, stigmasterol, lanosterol, fumaric acid, guanosine, N-acetyl-d-mannosamine, 3-hydroxybenzoic acid, citrulline, glycolic acid, chenodeoxycholic acid, serotonin, docosahexaenoic acid, Tyr, N-acetylglutamate, tartaric acid, Phe, homoserine, N-acetyl-5-hydroxytryptamine, glycerol 3-phosphate, quinic acid, spermine, L-cystathionine, L-glutamic acid, agmatine dehydroascorbic acid ↓; 2-ketobutyric acid, creatinine, (r)-3-hydroxybutyric acid ↑	Cirrhosis	L3 skeletal muscle area/heihgt2

&: cases/(cases+control) or cohort size; $: compared with non-sarcopenia or normal control, ↑: up-regulate,↓:down-regulate; *(+): positively associated with, (-): negatively associated with

Abbreviations: NMR: nuclear magnetic resonance; GC-MS: gas chromatography-mass spectrometry; CE-MS: capillary electrophoresis-mass spectrometry; LC-MS: liquid chromatography-mass spectrometry; ^31^P-MRS:31-phosphorus magnetic resonance spectroscopy; EWGSOP: European Working Group on Sarcopenia in Older People; FNIH: Foundation for the National Institutes of Health; AWGS: Asian Working Group for Sarcopenia; BA: bile acid; EPA: eicosapentaenoic acid; DHA: docosahexaenoic acid; ALM: appendicular lean mass; LPC: lysophosphatidylcholine; SM: sphingomyelin; PC: phosphatidylcholine; GPI: glycerol phosphsphorylinositol; GPC: glycerol phosphsphorylcholine; GPE: glycerol phosphsphorylethanolamine; ASM: appendicular skeletal muscle mass; BMI: body mass index; NEFA: Non-esterifed fatty acids; BCAA: branched chain amino acid; ALMI: appendicular skeletal muscle mass index; 12(S)-HETE:12-hydroxyeicosatetraenoic acid; 12(S)-HETRE:(12S)-hydroxy-(8Z,10E,14Z)-eicosatrienoic acid; AABA: a-aminobutyric acid;SDMA: symmetric dimethylarginine; Cr: creatinine; Gly: glycine; Ala: alanine; Val: valine; Leu: leucine; Ile: isoleucine; Pro: proline; Phe: phenylalanine; Tyr: tyrosine; Trp: tryptophan; Ser: serine; Thr: threonine; Met: methionine; Asn: asparagine; Gln: glutamine; Asp: aspartic acid; Glu: glutamic acid; Lys: lysine; Arg: arginine; His: histidine; the formula C X:Y was used to indicate the chain length as well as the number of double bonds; Diacyl or “aa” indicates that fatty acids are bound with ester bonds at the sn-1 and sn-2 positions on the glycerol backbone; Acyl-alkyl or “ae” indicates that the fatty acid at the sn-1 position is bound with an ether bond.

### Arginine, citrulline, polyamines

Arginine, a versatile amino acid, is considered a semi-essential amino acid vital for the human body. It is obtained through intestinal absorption from various food sources such as seafood, meats, nuts, seeds, watermelon, and soy [59]. Alternatively, it can be synthesized by the epithelial cells of the small intestine, kidneys, liver and other organs [60]. Arginine concentrations appear to be lower in the blood of sarcopenic patients compared to non-sarcopenic individuals, indicating potential alterations in arginine metabolism [61, 62] (Table 1). Aging skeletal muscle of rodents has shown increased activity of arginase, an enzyme involved in arginine catabolism, which can be induced by oxidative stress [63]. In animal experiments, dietary supplementation of arginine was found to up-regulate the expression of slow myosin heavy chain and down-regulate fast myosin heavy chain protein expression [64]. Additionally, it increased the levels of succinic dehydrogenase and malate dehydrogenase while decreasing the activity of lactate dehydrogenase. These findings suggest that arginine promotes a transition towards slow oxidative skeletal muscle fibers. Similarly, Stancic and colleagues discovered that arginine supplementation for a duration of 1 week in diabetic rats exhibited skeletal modifications akin to those observed with SOD mimic [65]. These alterations encompassed the up-regulation of the AMPK pathway, enhancement of mitochondrial function, and augmentation in the membrane translocation of glucose transporter 4(GLUT4) [64]. All the changes above were associated with mitigating the detrimental effects of diabetes on skeletal muscle, thereby suggesting that arginine may exert its action by ameliorating oxidative stress. However, multiple clinical studies have shown that short-term arginine supplementation does not yield significant improvements in skeletal muscle performance [66-68], with the exception of enhanced muscle blood perfusion observed in certain studies [68]. Furthermore, a 6-month dietary intervention study revealed improved transient muscle strength [69]. In conclusion, the role of arginine supplementation in skeletal muscle structure and function remains uncertain, and there is insufficient evidence to support its use as an enhancer for athletic performance.

Citrulline, one of the metabolites of arginine, is a non-essential amino acid that also appears to be specifically associated with sarcopenia [61, 70]. Unlike arginine, citrulline is not a protein building block and is not subject to catabolism by intestinal arginase and hepatic absorption [71, 72]. It therefore readily enters the circulation and subsequently reaches the kidneys to synthesize arginine. In 68 older adults over the age of 70, individuals with frailty and sarcopenia had higher levels of circulating citrulline [70] (Table 1). Likewise, another study by Duan et al found that 30 of 66 participating subjects with probable sarcopenia had higher plasma citrulline levels compared to controls [62]. In another cohort with combined viral hepatitis cirrhosis, plasma citrulline levels were lower in patients with sarcopenia than in the non-sarcopenia group [73]. The impact of citrulline on various exercise modalities is succinctly summarized in another comprehensive review [74]. Collectively, in the majority of interventional studies conducted on young, healthy individuals, it has been observed that the supplementation of arginine alone or in combination with other ingredients, along with exercise training, leads to an increase in circulating arginine concentrations and enhances the antioxidant capacity of skeletal muscles. These combined effects are commonly referred to as "ergogenic effects". The paradoxical effects of citrulline supplementation are observed in older adults with diminished nutrient absorption and metabolic capacity [75-78]. While the supplementation of citrulline has been shown to enhance arginine concentrations in the bloodstream [76], its impact on skeletal muscle performance necessitates further investigation. Notably, the influence of citrulline appears to be more pronounced subsequent to combined exercise training, suggesting its potential significance during periods characterized by heightened metabolic flux.

Polyamines, such as spermine and spermidine, play crucial roles in the growth and development of organisms by regulating cell proliferation/differentiation, immune response, autophagy induction, and metabolic regulation [79]. Derived from food, microbial and endogenous synthesis, polyamine levels gradually decline with aging and further decline in patients with sarcopenia [73]. Interestingly, Sanayama and colleagues made the intriguing observation that patients with sarcopenia exhibited higher levels of spermidine in their whole blood [80], despite a significant proportion of this cohort also experiencing comorbid cognitive decline, which is known to elevate circulating polyamine levels [81]. The exact effect of polyamines on skeletal muscle is still unclear, and numerous preclinical studies have been conducted to clarify this issue. Short-term spermidine administration was able to reactivate autophagy and improve fiber defects, whereas long-term feeding improved skeletal muscle function in *col6a1^-/-^* mice [82, 83]. The combination of spermidine and exercise synergistically activates the AMPK-FOXO3a signaling pathway, thereby inducing enhanced autophagy and upregulating anti-apoptotic capacity in D-gal-induced sarcopenia mice [84]. The supplementation of spermidine in C2C12 cells cultured *in vitro* demonstrated a mitigating effect on oxidative stress induced by H_2_O_2_, as well as the restoration of cell proliferation and migration [85]. These discoveries indicate that spermidine may potentially contribute to the process of injury repair and regeneration in skeletal muscle. Furthermore, polyamines exhibit the capacity to regulate gene expression by manipulating epigenetics, thereby highlighting their potential as a valuable tool in the field of molecular biology [86]. Overall, further investigation is warranted to fully comprehend the pathophysiological impact of polyamines on skeletal muscle, particularly considering the limited persuasive evidence from population studies. The implementation of research focused on biological efficacy and underlying mechanisms will undoubtedly contribute to a comprehensive understanding of sarcopenia's metabolic profiles and facilitate the development of effective interventions.

### Glutamine

Glutamine, a nonessential amino acid, is primarily synthesized, stored, and released by skeletal muscle. It serves as a vital energy source for various cells including immune cells, enterocytes, fibroblasts, and tumor cells [87]. Under certain circumstances, these cells show an increased dependence on glutamine compared to glucose. Multiple studies have consistently indicated an elevation in circulating glutamine levels among patients with sarcopenia [36, 43, 47, 88], with the exception of one which observed a down-regulation [62]. The discrepancy suggests a disruption in the metabolic activity of glutamine under sarcopenic conditions. Skeletal muscle protein homeostasis is disrupted in sarcopenia patients, characterized by aging-related anabolic resistance and proteolysis [12], which may promote glutamine synthesis, evidenced by upregulated glutamine synthetase activity uncovered in the rat [89]. Amirato and colleagues discovered that the oral administration of glutamine (10 g) to women aged over 60 for a duration of one month resulted in significant enhancements in knee muscle strength, power, and glycemic control, particularly when combined with exercise [90]. In another study, *ex vivo* myotubes exhibited a significant age-independent increase in both area and diameter 48 hours after the addition of glutamine [91]. This was followed by enhanced protein synthesis, as indicated by puromycin uptake, and activation of the mTOR signaling pathway. Notably, glutamine serves as a crucial energy source for immune cells. Moreover, sarcopenia is frequently accompanied by a persistent low-grade inflammatory state known as senescence-associated secretory phenotype (SASP) [92]. In response to this alteration, the elevation of glutamine levels may represent an adaptive mechanism.

## Lipids

Lipids constitute a diverse class of vital metabolites crucial for cellular viability, encompassing pivotal biological roles including energy provision, membrane architecture, and signaling. One of the histological characteristics of sarcopenia is the infiltration of lipids among myofibers, which involves the deposition of fat tissue and formation of intrafibrillar lipid droplets (IMCLs) [93], which suggests that lipid metabolism may undergo potential changes during aging and sarcopenia [94](Table 1). Lipids serve as reservoirs of energy in normal circumstances and can rapidly restore energy deficiencies in response to diverse signals such as hormones, nutritional status and physical activity. However, the excessive accumulation of lipids, especially in older adults who have limited physical activity or comorbid obesity, can lead to lipotoxicity [95].

### Fatty acids

With the advancement of metabolomics technology, an increasing number of studies are being conducted to elucidate the comprehensive lipid composition through lipidomics. Thanks to this cutting-edge technology, the enigma surrounding lipid metabolic components associated with sarcopenia is gradually being unveiled [96]. Excess dietary fatty acids are typically converted into neutral triglycerides, which are stored in adipose tissue. Upon receiving catabolic stimuli such as starvation and exercise, triglycerides undergo sequential hydrolysis by adipose triglyceride lipase (ATGL), hormone-sensitive lipase (HSL) and monoglyceride lipase (MGL) to liberate free fatty acids into the circulation [97]. Fatty acids are transported into myocytes through various mechanisms, including muscle lipoprotein lipase (mLPL), cluster of differentiation 36 (CD36), fatty acid banding proteins (FABPs) and fatty acid transport proteins (FATPs) [98]. Upon entry into the cytosol, fatty acyl-CoA synthetase (ACS) initiates the activation of free fatty acids. These activated fatty acids are then efficiently transported to the mitochondria for β-oxidation [98].

Notably, Dalle and colleagues observed a significant elevation in circulating oxylipins derived from polyunsaturated fatty acids among patients with sarcopenia [99]. This finding suggests that the excessive presence of oxylipins may impact sarcopenia by modulating inflammatory responses [29]. In several other studies, there were significant reductions or positive correlations observed between circulating polyunsaturated fatty acids and muscle performance in patients with sarcopenia [73, 100-102]. Conversely, saturated fatty acids exhibited adverse associations with muscle strength/mass/function, particularly long-chain fatty acids [39, 103]. Studies have primarily concentrated on investigating the muscle modulatory effects of n-3 and n-6 polyunsaturated fatty acids due to their recognized anti-inflammatory properties, promotion of protein synthesis, and ability to combat insulin resistance [24, 104]. A recent comprehensive review conducted by Huang extensively elucidated the pivotal role of polyunsaturated fatty acids (PUFAs), highlighting that over half of the clinical studies unequivocally demonstrated their positive effects [105]. Furthermore, synergistic benefits were observed when PUFAs were combined with exercise, suggesting a potentially enhanced efficacy in promoting health outcomes. Possible mechanisms of action, such as the modulation of the autophagy-proteasome system, attenuation of oxidative stress, and enhancement of insulin sensitivity, have also been investigated in PUFA-interventional clinical and preclinical studies [106-108]. It is crucial to acknowledge that lipid composition exhibits a high level of complexity, and metabolites can undergo significant alterations in response to even minor environmental perturbations. Therefore, it is imperative to conduct more rigorously designed studies with larger sample sizes in order to obtain more precise metabolic landscapes and facilitate more comprehensive mechanistic investigations.

### Phospholipids

Phospholipids, beyond their role in constituting cellular and organelle membranes, also serve as a crucial class of signaling molecules. As such, the investigation of phospholipids has shifted towards comprehending their impact on protein structure and function, a field that garners significant attention in contemporary research [109]. Glycerophospholipids are the predominant components of biological membranes and include the commonly encountered phosphatidylcholine, phosphatidylethanolamine, phosphatidylglycerol, phos-phatidylinositol and phosphatidylserine [110]. Kemp and Hinkley have identified elevated levels of glycerophospholipids in blood and skeletal muscle conducted on individuals experiencing muscle loss, respectively [111, 112]. These findings suggest that the process of muscle loss may be accompanied by modifications in the lipid composition of myocyte membranes, potentially linked to a reduction in type II muscle fibers. Notably, Lysophosphatidylcholine 18:2 consistently demonstrated a positive correlation with muscle loss or frailty and displayed a significant decrease across all three studies [39, 50, 113]. This consistent pattern positions it as a promising biomarker for hypokinesia prediction. Plasma sphingomyelins (SM) are also vital constituents of cellular membranes and exhibit an inverse association with grip strength (SM C20:2, SM C18:1, SM C16:1, SM C18:0, and SM (OH)C22:2), as well as gait speed (SM C18:1, SM C16:1, SM C18:0, and SM C20:2) [113, 114]. While the concrete molecular mechanisms underlying these aforementioned phospholipids remain to be thoroughly investigated, a multitude of preclinical studies indicate that these phospholipid molecules may potentially play a role in processes such as inflammation, insulin sensitivity, and manipulation of mitochondrial function [114-116]. This is further reflected in various cardiovascular and neurological disorders [110]. Significant alterations in the composition and structure of membrane phospholipids are indicative of physiological adaptations and stress responses within cells and organelles. Deciphering these mechanisms could potentially offer a novel approach to addressing sarcopenia.

### Oxysterols

Oxysterols, a class of oxidation products derived from cholesterol through enzymatic or non-enzymatic pathways, have been found to be involved in many age-related diseases [117]. The most extensively studied is their presence in atherosclerotic plaques, where they are a major component [118, 119]. Further *in vitro* and *in vivo* studies have confirmed that these steroid derivatives with oxygenated polar groups can be involved in a variety of diseases, involving mechanisms such as immune regulation, inflammation, oxidative stress [120], affecting cell survival, proliferation, and differentiation [121]. Similarly, in the study of sarcopenia, research has found that these unique oxidized sterols may also play a significant role that cannot be ignored. For instance, Liu discovered a significant decrease in plasma lanosterol levels among patients with sarcopenia [73]. Several recent studies have also reported increased plasma levels of 7β-hydroxycholesterol and 7-ketocholesterol, the products of cholesterol auto-oxidation, in individuals with sarcopenia [122]. Furthermore, they found that both oxysterols induced cell death in differentiated and undifferentiated C2C12 cells *in vitro* and promoted the release of inflammatory factors such as TNFα and IL-8, which are involved in the mechanism of oxidative apoptosis (oxiapoptophagy) [122, 123]. 20(S)-hydroxycholesterol has likewise been shown to inhibit chicken satellite cell proliferation and differentiation [124]. Muscle waste was observed in wild-type mice injected intraperitoneally with 25-hydroxycholesterol, whereas TNF alpha type 1 receptor knockout (TNFR1KO) mice did not undergo such alterations, suggesting that 25-hydroxycholesterol can play a role by inhibiting the IGF -1/Akt signaling pathway [125]. In conclusion, the role of oxidized sterols in skeletal muscle is gradually being revealed, however, more *in vivo* and *in vitro* studies are needed to further confirm this effect.

## Glucose

Skeletal muscle is responsible for the distribution of most postprandial glucose [126]and is essential for maintaining glucose homeostasis in the body through insulin/IRS/GLUT4-mediated uptake of glucose [127]. There is a structural and functional basis for impaired glucose utilization in aging and sarcopenia, leading to a subsequent decline in skeletal muscle performance. Skeletal muscle, as glucose distribution sites, has a reduced ability to maintain glycemic homeostasis when muscle mass is reduced [27, 128]. This may be partly attributed to a reduced proportion of type II muscle fibers and a diminished number of type I fibers. As demonstrated by Uchitomi and colleagues, the levels of fructose 1,6-diphosphate and dihydroxyacetone phosphate in skeletal muscle were significantly reduced in 28-month-old aged mice compared to their 8-week-old young counterparts [129]. This observation suggests a potential decline in glycolytic capacity. Not only that, but several studies have also indicated an age-related decline in both the quantity and functionality of mitochondria in skeletal muscle [130-133]. Mitochondria play a crucial role in the catabolism of glucose via oxidative phosphorylation to generate ATP, which is indispensable for sustaining diverse cellular activities. This regression of mitochondria can be attributed to impaired PGC-1α/ERRα signaling, disrupted mitochondrial dynamics (division, fusion, and autophagy), altered gene and protein expression, reduced ATP production and increased reactive oxygen species (ROSs) production within sarcopenic muscle. Consistently, in conjunction with the aforementioned modifications, lipid deposition within the muscle fibers facilitates the accumulation of intracellular lipid derivatives such as ceramides and diacylglycerol [134]. This process leads to the activation of protein kinase C (PKC), inhibition of IRS-1 tyrosine phosphorylation and PI3-kinase activity, ultimately resulting in insulin resistance [134, 135]. Meanwhile, the presence of interfibrillar lipids stimulates the secretion of inflammatory cytokines, leading to the development of a chronic low-grade inflammatory microenvironment [136]. Advanced glycosylation end products are a collection of nonenzymatic adducts that inevitably form as an inherent part of the aging process. It is believed that these AGEs play a role in mediating insulin resistance by inducing endoplasmic reticulum stress through the generation of reactive oxygen species [137, 138]. Collectively, glucose serves as a crucial metabolic substrate for skeletal muscle, while the aging-induced decline in anabolism and degenerative changes impede metabolic flexibility. This highlights its potential as a viable target for therapeutic intervention.

## Carnitine, acylcarnitines

Carnitine and acylcarnitines form the carnitine pool in organisms. The former acts as a cofactor, facilitating the transportation of free fatty acids from the cytoplasm to the inner mitochondrial membrane for β-oxidation [139]. On the other hand, the latter serves as an intermediate product obtained through fatty acid exchange between carnitine and acyl-coenzyme A (acyl-CoA). Carnitine levels serve as an indirect indicator of mitochondrial metabolic activity, and recent research has further substantiated its potential as a nutritional supplement for enhancing muscle function [140]. According to Lo and colleagues, plasma carnitine and multiple acylcarnitines were found to be elevated in individuals with sarcopenia and severe sarcopenia. Furthermore, after adjusting for various confounding factors, butyrylcarnitine remained significantly associated with low physical function and the presence of sarcopenia [141]. These findings suggest a potential comorbidity between muscle loss and mitochondrial fatty acid metabolism. In a previous study, there was an observed elevation of plasma acetylcarnitines levels in patients undergoing arterial surgery and experiencing rapid muscle mass loss [111]. Similarly, patients with limited mobility who had been bedridden for 6 months showed similar results [142]. Interestingly, in a study involving 430 early postmenopausal women, serum L-carnitine was found to be positively correlated with appendicular lean mass (ALM) and ALM/height2 (β=0.448, p=0.015; β=0.184, p=0.004) [143]. Conversely, acetylcarnitines showed a negative correlation with these variables [143]. However, it remains uncertain whether this change is influenced by sex hormones. Nevertheless, it has been observed that frail older people have a significant decrease in both skeletal muscle carnitine acyltransferase mRNA and enzyme activity compared to their healthy counterparts [144-146], suggesting that the aforementioned changes in circulating acylcarnitine levels may stem from either a deficiency in mitochondrial oxidative capacity or an altered substrate selectivity within the mitochondria. Given the intimate association between carnitine and mitochondria, a series of investigations have delved into the correlation between carnitine supplementation and muscle mass and function. These studies showed that carnitine supplementation significantly increased both circulating and skeletal muscle carnitine levels, resulting in a concomitant increase in lipid oxidation [147, 148]. In another intervention study conducted on older women, 24-week supplementation of L-carnitine combined with leucine did not yield significant improvements in resistance exercise training [149]. Further clinical and basic research is needed to clarify the exact role of carnitine/acylcarnitines.

## Microbiota

The gut microbiota colonizes the gastrointestinal tract with trillions of bacterial cells and is believed to interact with host disease and health through modifications in composition and metabolism involving immunity, energy and neurological disorders [150]. In recent years, there has been a growing research focus on exploring the relationship between the gut microbiota and its metabolic activity in relation to skeletal muscle [151] (Fig. 1). These investigations aim to provide insights into the underlying causes of sarcopenia and potential intervention strategies by examining the gut microbiota-muscle axis. With the improvement of research tools, there has been a significant improvement in comprehending intricate microbiome profiles. This progress enables an extensive exploration of microbial metabolites from phenotype to function, thereby offering additional perspectives for comprehensive sarcopenia analysis.

### Short-chain fatty acids

Short-chain fatty acids, derived primarily from carbohydrates that are resistant to intestinal digestive enzymes and fermented by colonic microbes, serve as an energy source for these microbes and help maintain the unique environment within the intestinal lumen [150]. A series of cross-sectional studies have revealed that patients with sarcopenia exhibit a reduced abundance of gut microbiota capable of producing short-chain fatty acids (SCFAs) in their feces, as well as diminished production of SCFAs [152-157]. Preclinical investigations have demonstrated that supplementing mdx mice with sodium butyrate for a duration of 3 weeks can yield motility-preserving effects comparable to deflazacort (DFZ), partly by restoring autophagic capacity and exerting anti-inflammatory effects in mdx mice [158]. Furthermore, in aged sarcopenic mice exhibiting gut microbial dysbiosis, there was a significant reduction in the number of satellite cells and a loss of quiescence [159]. However, supplementation with butyrate reversed the antibiotic-induced activation of satellite cells and aided in maintaining their stemness by inhibiting both myoblast proliferation and differentiation [159]. Liu et al. demonstrated that butyrate exerts a beneficial effect on muscle atrophy in malignant mice by modulating the Akt/mTOR/Foxo3a and Fbox32/Trim63 signaling pathways, as well as facilitating the transition of macrophage polarization from M1 to M2 type [160]. Consistently, in an *in vitro* culture system with a concentration of 750μM butyric acid, C2C12 cells exhibited enhanced cell proliferation through the activation of the mitogen-activated protein kinase (MAPK) signaling pathway [157]. These *in vivo* and *in vitro* studies have demonstrated the potential to enhance bacterial colony structure and metabolic components for intervening in sarcopenia. However, further research is required to validate the transferability of these effects to humans.

### Bile acids

Primary bile acids, metabolites of cholesterol, are synthesized by hepatocytes and include bile acids and goose deoxycholic acid, which are secreted with bile into the intestinal tract, where they are converted to secondary bile acids by the bile salt hydrolase of gut microbes [161]. Secondary bile acids metabolized by distal gut microbes are able to enter systemic circulation, creating a link between gut flora and skeletal muscle. Bile acids not only facilitate the intestinal uptake of lipids, but also serve as ligands for various nuclear receptors, such as farnesoid X receptor (FXR), which mediate intercellular and interorgan signaling [162]. In patients with sarcopenia combined with decompensated cirrhosis, elevated levels of serum secondary bile acids such as deoxycholic acid, lithocholic acid, deoxycholic acid/cholic acid ratio, and lithocholic acid/goose deoxycholic acid ratio indicate an increased microbial transformation in sarcopenic individuals [41], suggesting a possible alteration in the liver-intestinal flora-skeletal muscle axis. Indeed, Qiu et al. conducted preclinical experiments and observed a significant reduction in both intestinal FGF15 and FXR levels in aged sarcopenic mice [163, 164]. Additionally, the researchers found that antibiotic inactivation of intestinal microbiota led to pronounced muscle atrophy accompanied by an inhibition of the bile acid signaling pathway known as Ileal farnesoid X receptor (FXR)-fibroblast growth factor 15 (FGF15) [163, 164]. These findings suggest a potential association between bile acid deficiency and muscle atrophy. Similarly, Sun et al. discovered that lithocholic acid supplementation enhanced the repair of skeletal muscle injuries and demonstrated *in vitro* that lithocholic acid can activate signaling for protein synthesis while inhibiting protein degradation through the TGR5 receptor (also known as G protein-coupled receptor 1, GPBAR1)/AKT/mTOR /FoxO3 cascade [165]. Interestingly, based on a series of studies conducted by Abrigo et al., it has been observed that cholic acid and deoxycholic acid can induce muscle atrophy in mice through a TGR5 receptor-dependent mechanism, specifically by causing mitochondrial dysfunction in skeletal muscle [166-168]. The aforementioned conflicting findings imply that the association between bile acids and skeletal muscle might be influenced by multifaceted factors, including the impact of colony structure. Further investigations are warranted to elucidate this relationship.

## Strategies for modulation of metabolism to counteract sarcopenia

Despite extensive research efforts, the current state of treatment for sarcopenia remains ineffective [169, 170]. Given that age-related muscle mass loss is an inevitable outcome, there is a growing clinical significance in exploring strategies to decelerate this process or enhance overall bodily function. As previously mentioned, the cascade of age-related metabolic alterations has prompted us to explore feasible interventions aimed at mitigating the aging progression and attenuating muscle mass decline through a modulated metabolic perspective. Consequently, the subsequent section of this review thus concentrates on intervention strategies that may encompass metabolic alterations and are concisely delineated below.

Lifestyle interventions are frequently deemed safe, cost-effective, and scientifically validated, making them an essential component of prescription recommendations. As previously mentioned, supplementation with nutrients is beneficial in preventing muscle mass loss and promoting functional recovery. Whey protein, branched-chain amino acids, vitamin D, and β-hydroxy-β-methyl butyrate (HMB) can all serve as dietary intervention options, with their muscle/bone benefits being substantiated by a series of clinical studies [169]. Given the interplay between gut microbiota and skeletal muscle, probiotics, as bacterial ecological modifiers, can be considered a viable therapeutic option for patients experiencing imbalances in gut flora homeostasis [171, 172]. Exercise is an essential and efficacious strategy in enhancing physical fitness. Aerobic exercise proves more advantageous in improving athletic performance and cardiorespiratory endurance, while resistance exercise aids in developing muscle strength. The incorporation of progressive resistance training, alongside balance training, may potentially yield superior outcomes [173].

Lifestyle interventions may pose challenges in patients with functional limitations, necessitating the development of pharmacologic therapies. As previously mentioned, numerous studies have revealed a frequent coexistence of lipid metabolism disorders in sarcopenia. Furthermore, the accumulation of abnormal lipids within myocytes' intracellular/interstitial spaces exacerbates insulin resistance, inflammation, and lipotoxicity. Supplementation with polyunsaturated fatty acids, including alpha-linolenic acid (ALA), eicosapentaenoic acid (EPA), docosapentaenoic acid (DPA), and docosahexaenoic acid (DHA), may effectively combat aging-associated inflammation, promote skeletal muscle protein synthesis, reduce protein degradation, and improve mitochondrial function [174]. These strategies possess a favorable safety and tolerability profile while increasing cellular amino acid uptake and improving cell membrane fluidity. Therefore, they hold promises as cost-effective approaches for restoring muscle performance. FTY720, a sphingosine-1-phosphate (S1P) analog, competes for binding to the S1P receptor, downregulates its expression, and reduces interstitial accumulation of ceramides and sphingolipids [175]. However, it does not significantly impact insulin sensitivity. In response to elevated oxysterols, pexotherapy such as tocopherols and milk thistle seed oil have been shown to attenuate oxysterol-induced oxidative stress in C2C12 cells [123, 176]. Testosterone supplementation in older men with decreased physiological levels can enhance lean body mass and lipid oxidizing capacity without exerting a significant effect on insulin sensitivity [93, 177].

Maintaining glucose metabolic homeostasis is crucial for preserving normal muscle mass, as glucose serves as the primary energy source for skeletal muscle. In light of the increased risk of comorbid sarcopenia in elderly individuals, potential effects on skeletal muscle should be taken into consideration when administering glucose-lowering medications. Metformin, an AMPK-targeting insulin sensitizer, theoretically enhances insulin sensitivity in skeletal muscle; nevertheless, its effects on skeletal muscle in clinical practice remain a subject of controversy [178]. Thiazolidinediones are agonists of peroxisome proliferator-activated receptor gamma (PPARγ), which enhance fatty acid metabolism in skeletal muscle and potentially contribute to the preservation of muscle mass, regardless of resistance exercise [179]. SGLT-2 inhibitors, known for their ability to inhibit sodium-glucose cotransportation and reabsorption in the proximal renal tubule, have yielded conflicting results in both preclinical and clinical studies [180]. GLP-1 analogues and DPP-IV inhibitors, conversely, have been observed to mitigate muscle mass loss, while flavonoids and glinides may potentially induce a decline in muscle mass [178]. Glucose-lowering medications are extensively utilized among the elderly demographic, necessitating careful consideration of potential skeletal muscle implications during the development of pertinent therapeutic protocols. In addition, mitochondria, the central hub of skeletal muscle metabolism, undergo structural, quantitative, and functional alterations during aging and sarcopenia. Numerous clinical and preclinical studies have been dedicated to reversing or preserving mitochondrial metabolic homeostasis in order to enhance skeletal muscle quality. Mitochondrial dysfunction and chronic inflammation both contribute to the development of insulin resistance and anabolic resistance. Resveratrol, a naturally occurring polyphenol derived from plants, has been shown to effectively reverse mitochondrial dysfunction by activating the AMPK signaling pathway. Additionally, it has demonstrated the ability to enhance muscle function and increase muscle mass in both mice and humans [181, 182]. Therefore, resveratrol holds great potential as a promising therapeutic agent for myasthenia gravis treatment.

## Concluding remark

Aging is a natural physiological process, and skeletal muscle atrophy is one of the systemic changes linked to aging, which impairs patients' ability to withstand various stimuli. Simultaneously, the process of aging is accompanied by a reduction in overall metabolic flexibility, consequently leading to remodeling of skeletal muscle metabolism. Metabolomics technologies, which involve the identification and quantitative analysis of small molecules known as metabolites, provide a more detailed depiction of the cellular metabolic landscape. This enables us to visualize real-time and dynamic changes in cellular metabolic activities with greater precision. As a consequence, an increasing number of pathophysiologically relevant small molecule metabolites have been identified. Metabolites serve as indicators of metabolic activities and can reflect the aberrant functioning of diverse proteins within the metabolic pathway. Moreover, the abnormal accumulation of metabolites may exert an impact on cellular, organelle, and even protein structure and functionality. Detection of absolute or relative levels of metabolites provides insights into physiological or pathological changes, adjustment of abnormal metabolite levels can aid in reversing metabolic consequences, and modification of naturally occurring active metabolic components may facilitate the development of therapeutic drugs. These observations suggest that functional metabolomics will be a pivotal direction for future advancements. Metabolic investigations on age-related sarcopenia have offered novel insights and clues for comprehending the disease. However, given the susceptibility of natural metabolites to perturbations, accurately reflecting the body's condition through metabolic composition analysis remains a concern. Perhaps an integrated multi-omics analysis encompassing genomics, transcriptomics, proteomics, epigenomics, and macrogenomics could offer enhanced sensitivity and a comprehensive profile for precise comprehension of sarcopenia pathogenesis.
